# Tracheal Cartilage Fracture: A Rare Cause of Weaning Failure

**DOI:** 10.7759/cureus.63297

**Published:** 2024-06-27

**Authors:** Sanjay Gabhale, Sidhaant Nangia, Hiral Ramnani, Mithun Nilgiri K

**Affiliations:** 1 Respiratory Medicine, Dr. D. Y. Patil Medical College Hospital and Research Centre, Dr. D. Y. Patil Vidyapeeth, Pune (Deemed to be University), Pune, IND

**Keywords:** obstructive ventilatory defect, fiber optic bronchoscopy, post tracheostomy tracheal stenosis, weaning failure, tracheal ring fracture

## Abstract

A 42-year-old female developed a rare complication of tracheal ring fracture following repeated percutaneous dilatational tracheostomy, which was performed after intubation due to progressive respiratory failure in the case of treated organophosphate poisoning. The patient first presented with organophosphate poisoning and was intubated in view of altered sensorium and tracheostomized after a prolonged stay in the intensive care unit. The patient was successfully weaned off and the tracheostomy tube was removed; the patient had progressive breathlessness over the duration of five months and presented with stridor, requiring emergency intubation and repeat tracheostomy due to respiratory failure. Imaging studies showed bilateral pleural effusion, right middle lobe consolidation, and scattered ground glass opacities. The patient received intravenous antibiotics and fluid therapy but faced challenges with weaning despite meeting the criteria. Bronchoscopy revealed a broken tracheal cartilage obstructing the tube, which was removed, leading to improved respiratory status and successful weaning off the ventilator. The patient underwent tracheal wall repair, was decannulated, and discharged successfully following extubation.

## Introduction

Tracheal ring fracture is rare but a known complication of percutaneous dilatational tracheostomy (PDT), it is an iatrogenic injury post-tracheostomy, which is influenced by the method of tracheostomy and the pressure applied during the procedure. Trauma to the trachea causes inflammation and granulation tissue formation, which can further lead to tracheal stenosis, causing symptoms like shortness of breath, stridor and obstructive ventilatory failure [1]. Diagnosis of tracheal stenosis is usually done by bronchoscopy, which is routinely done post-tracheostomy to assess airway patency, identify post-tracheostomy complications, and clear any obstructing tissue [2] and surgery is the main therapeutic intervention in patients with post-tracheostomy tracheal stenosis (PTTS) [3], where the stenotic area is surgically resected and ends are anastomosed; however, in cases where surgery is not suitable, bronchoscopy guided interventions provide a less invasive option as alternative therapeutic management [4]. PTTS can present at different sites within the trachea, such as at the stoma, site of the cuff, or distal tip of the tracheostomy tube [5] and factors like infection, chondritis, cuff pressure, or prolonged impingement of the tracheal wall by the tube tip can cause stenosis [2,5]. While treatment strategies for PTTS lack well-defined guidelines or rigorous trials, a multidisciplinary approach involving otolaryngologists, interventional pulmonologists, and thoracic surgeons is essential for successful management [4]. Surgical resection is effective but carries risks of morbidity and mortality, including restenosis, granuloma formation, infections, and bleeding [3]. Bronchoscopy is often utilised preoperatively to evaluate the airway and aid ventilation during surgery [5], in emergencies like acute airway obstruction, where surgery may not be feasible, interventional bronchoscopy can be a viable alternative [6].

## Case presentation

**Figure 1:**
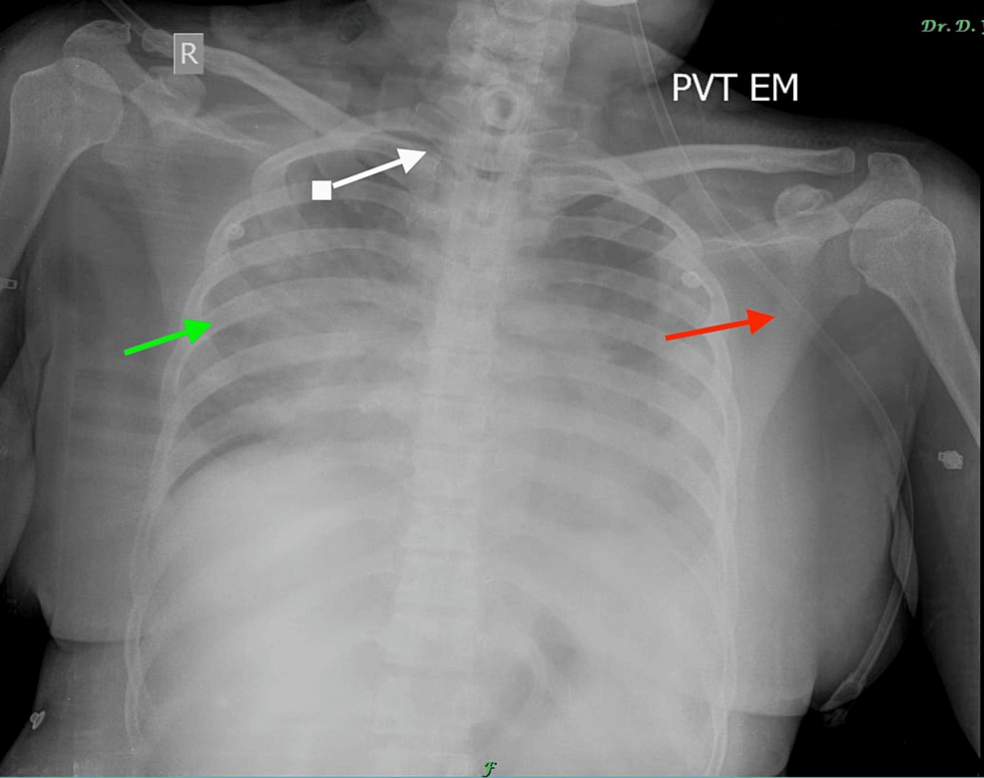
Chest radiograph (anteroposterior view) showing tracheostomy tube in situ (white arrow), nasogastric tube (red arrow) in situ, with right upper lobe consolidation (green arrow) and bilateral cardiophrenic angle blunting. PVT EM: Private Emergency Medicine

A 42-year-old female presented to the emergency with stridor and desaturation, intubated and tracheostomised from outside. The patient had a history of organophosphate consumption and was previously intubated and tracheostomised, because of respiratory failure, five months back. After one month, the patient was symptomatically better, met the weaning criteria, was weaned off the ventilator and decannulated. The patient’s bronchoscopy was done which revealed granulation tissue, that bled on touch and the stoma was closed and the patient was discharged after two days with inhaled bronchodilators and a short course of oral steroids. The patient was asymptomatic for four months, after which she presented to another outside hospital with breathlessness in a drowsy and disoriented state, where she was intubated because of a low score on the Glasgow Coma Scale (GCS) and subsequently tracheostomised after 10 days of mechanical ventilation. The patient was having difficulty weaning, suspecting acute airway obstruction; a contrast-enhanced computed tomography (CECT) scan of the neck was done, which showed no endotracheal stenosis or mass. The patient was sent to our casualty with a tracheostomy tube in situ with ventilatory failure, stridor and hypoxia despite receiving a 100% fraction of inspired oxygen (FiO2). Subsequent chest radiograph revealed bilateral lower lobe collapse with consolidation (Figure 1).

**Figure 2:**
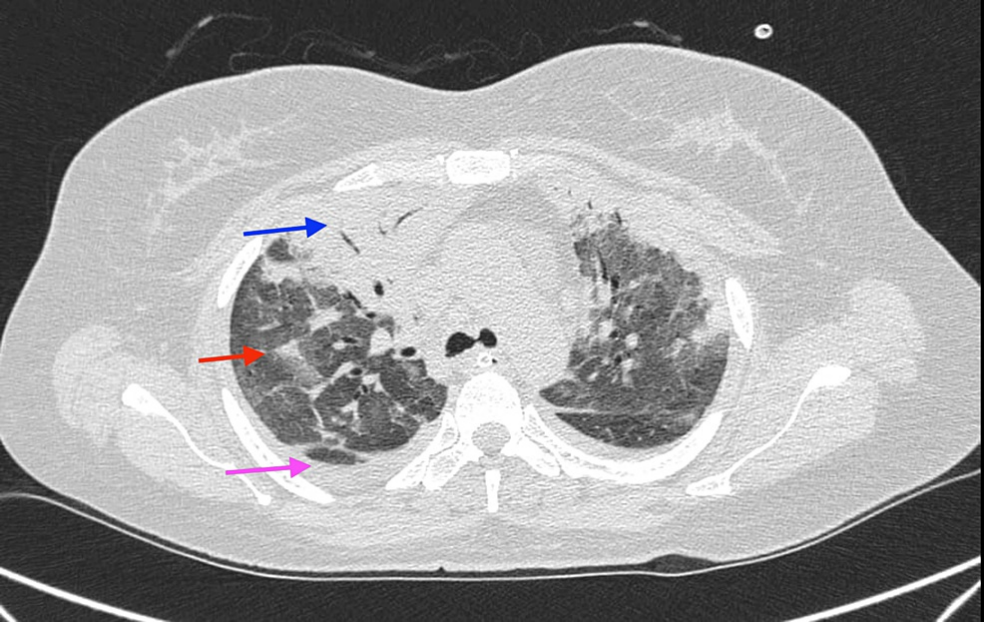
Computed tomography (CT) scan of thorax (coronal view) showing right upper lobe consolidation (blue arrow), extensive ground glass opacities (red arrow), bilateral moderate pleural effusion (pink arrow).

**Figure 3:**
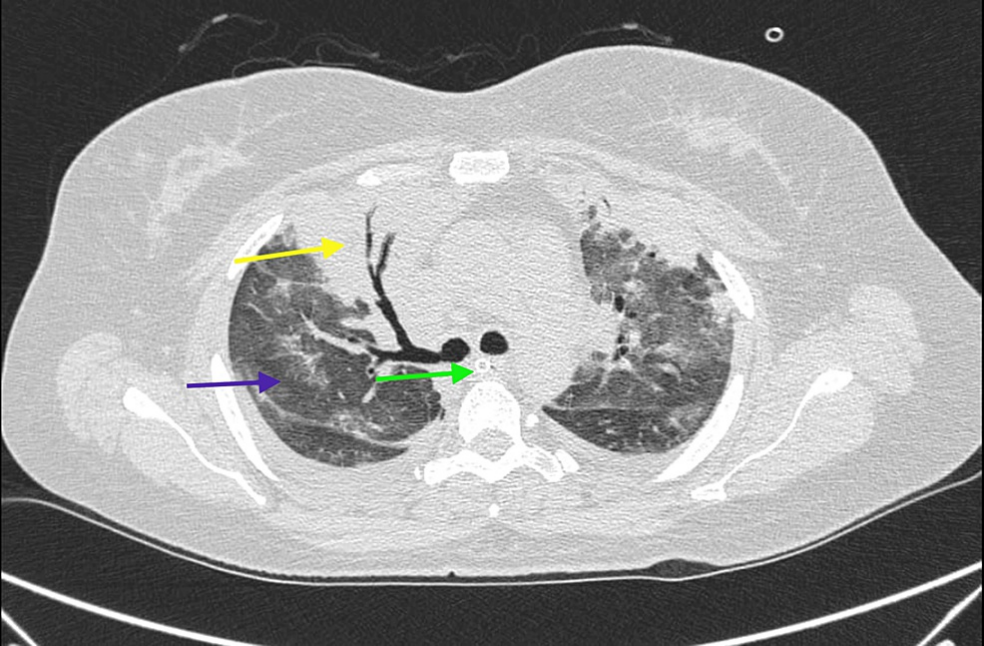
Computed tomography (CT) scan of thorax (coronal view) showing right upper lobe consolidation with air bronchogram (yellow arrow), extensive ground glass opacities with fissural effusion (purple arrow) and tracheostomy tube in situ (green arrow).

Arterial blood gas analyses indicated type 2 respiratory failure and routine blood investigations showed raised total leukocytic counts, apart from that, the rest of the parameters were normal. The patient was subsequently admitted to the intensive care unit (ICU) on the volume control mode of ventilation with 100% FiO2 via the tracheostomy tube, intravenous antibiotics, fluid replacement therapy and nebulisation with long-acting bronchodilators and inhaled corticosteroids were initiated. For further evaluation, a CECT thorax was done, which revealed bilateral moderate pleural effusion with dense consolidation in the right middle lobe and the right upper lobe with extensive ground glass opacities and no air trapping, the pre- and para-tracheal spaces were normal with a tracheostomy tube in situ (Figures 2, 3).

**Figure 4:**
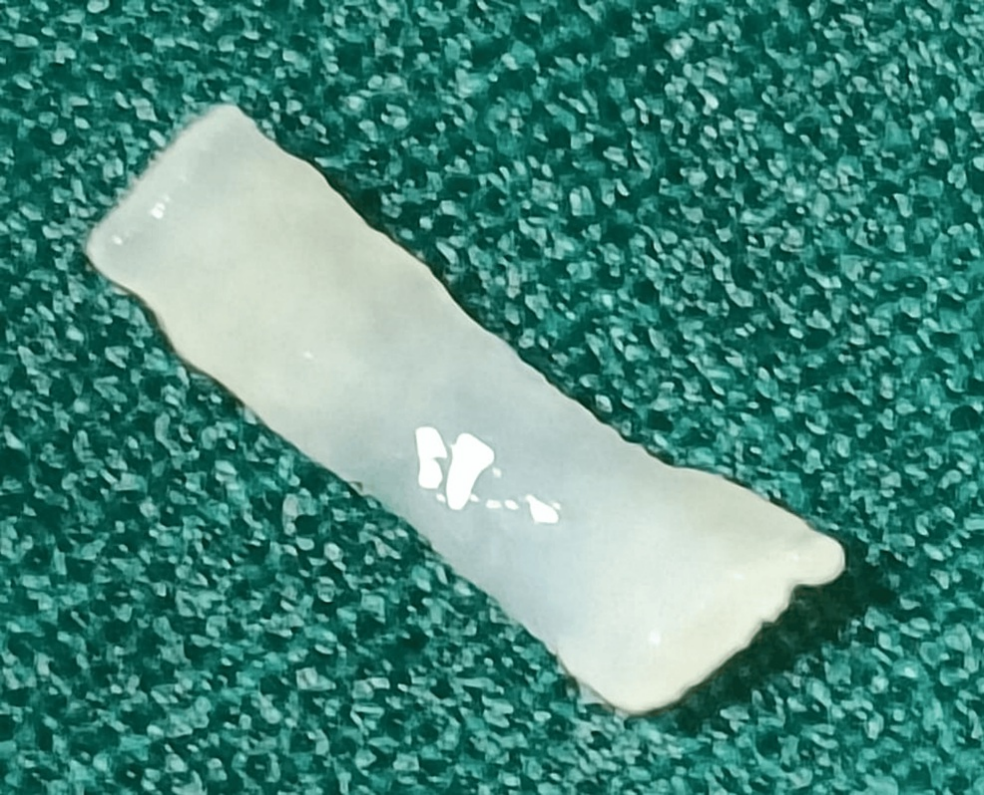
Broken fragment of the tracheal ring removed via the bronchoscope.

The endotracheal secretions were sent for culture but didn't isolate any organism. Due to continued stridor despite nebulisation, tracheal stenosis was suspected and a check bronchoscopy was done. The check bronchoscopy revealed a broken cartilage, which was an incidental finding, obstructing the tracheostomy tube with narrowing of the tracheal lumen. The broken cartilage was subsequently removed and the patient improved symptomatically; the stridor was reduced and the oxygen requirement came down (Figure 4).

After hemodynamic stabilization, the patient was weaned off the ventilator and kept on oxygenation therapy with intermittent non-invasive ventilation (NIV). Within a few days, the patient’s ventilatory demand was reduced and the patient was weaned off NIV with no supplemental oxygen requirement. After decannulation, the tracheal wall was repaired and sutured and as soon as the underlying consolidation was resolved, the patient was successfully discharged from the hospital.

## Discussion

Tracheostomy is an effective airway management for patients who are on mechanical ventilation for a prolonged period, extending more than 10-14 days [7]; it has been shown to reduce stay in the ICUs, ventilator-associated pneumonia and complications of prolonged intubation like subglottic stenosis [8,9]. Even though PDT is widely accepted as the procedure of choice for tracheostomy, it can lead to severe complications including even death, annually, about 500 patients in the United States die or are permanently disabled due to tracheostomy [10]. A meta-analysis of 8,324 PDT cases estimated a procedure-related mortality rate of 2.18%, with 31% of these being intra-procedural fatalities and 49% occurring within seven days of the procedure [11], and late complications occur in up to 65% of patients [12], this can further lead to tracheal pseudo membrane formation, composed mainly of necrotic epithelium and fibrin, which may cause tracheal stenosis. Around 3-12% of patients with tracheal narrowing due to tracheostomy develop clinically significant stenosis needing intervention [13]. Early signs manifest as a weaning failure, high peak airway pressures, or the suction tube failing to pass via the tracheostomy tube, later it may present as decannulation failure, upper-airway obstruction, dyspnoea, stridor or even respiratory failure after decannulation [12]; these symptoms of stenosis typically arise when the tracheal lumen narrows to less than 50% of its original diameter, with exertional dyspnoea occurring at less than 10 mm and stridor at less than 5 mm [14].

Multiple factors predispose patients to develop PTTS like a high tracheostomy site, trauma during intubation, prolonged intubation, history of tracheostomy or intubation, excessive corticosteroid use, elderly, female sex, obstructive sleep apnoea, and targeted radiotherapy for oropharyngeal and laryngeal carcinoma [15,16]. PTTS can occur at multiple sites within the trachea, including the cuff site, stoma or distal tip of the tracheostomy tube and traumatic injury during the procedure can lead to stenosis by rupturing or displacing tracheal rings, resulting in "A-shaped" strictures [5], even though it is a common complication, treatment is not well defined, with no rigorous randomised controlled trials or established guidelines to help in management [4]. Surgical resection followed by end-to-end anastomosis is the usual treatment for symptomatic PTTS [3]; however, this approach has notable morbidity and mortality rates, with mortality rates reported as much as 1.8% and morbidity including restenosis, granuloma formation, infections, and bleeding, which is seen in up to 14% of the cases, further, cricoid or thyroid cartilage anastomosis has shown poorer wound healing [3]. Bronchoscopy is routinely used pre-surgery to evaluate the airway and look for any obstruction preventing effective ventilation [2], in cases like subglottic stricture, surgery may be contraindicated due to severe comorbidities, in such cases, interventional bronchoscopy provides a less invasive treatment option with success rates up to 66% [6]. In instances like our case, bronchoscopy can go beyond a tool for pre- and post-tracheostomy airway evaluation and can be an effective therapeutic intervention for cases like severe tracheal stenosis, tracheal obstruction due to dislodged tracheal cartilage post-traumatic tracheostomy, recurrent tracheal stenosis and tracheomalacia.

## Conclusions

Percutaneous tracheostomy is a widely performed procedure in the ICUs, indicated in patients on the mechanical ventilator for a prolonged period; it is generally considered safe but has some procedural complications. Complications like trauma to the trachea leading to tracheal stenosis are mainly iatrogenic, performers should be careful to avoid it and further know how to identify it early, to minimise mortality and morbidity. Currently, surgery followed by end-to-end anastomosis is the main treatment and bronchoscopy is routinely used for pre- and post-tracheostomy airway evaluation; in this case, we show that bronchoscopy can be an effective and less invasive interventional management for PTTS.
